# Incidence and characteristics of distal radius fractures in a southern Swedish region

**DOI:** 10.1186/1471-2474-8-48

**Published:** 2007-05-31

**Authors:** Elisabeth Brogren, Michael Petranek, Isam Atroshi

**Affiliations:** 1 Department of Orthopedics Hässleholm-Kristianstad, Hässleholm Hospital, SE-29125 Hässleholm, Sweden; 2 Department of Radiology, Hässleholm Hospital, SE-29125 Hässleholm, Sweden

## Abstract

**Background:**

The incidence of distal radius fracture has increased substantially during the last 50 years according to several studies that estimated the overall incidence in various general populations. The incidence of fracture classified according to severity has not been well documented. The aim of this population-based study was to estimate the overall and type-specific incidence rates of distal radius fracture in a representative population in southern Sweden.

**Methods:**

During 2001, all persons older than 18 years with acute distal radius fracture in the southern Swedish region of Northeastern Scania were prospectively recorded. A radiologist classified the fractures according to the AO system and measured volar tilt and ulnar variance. A fracture with volar tilt outside a range of -5° to 20° and/or ulnar variance of 2 mm or greater was defined as displaced.

**Results:**

335 persons with acute distal radius fracture were recorded during the 1-year period. The overall incidence rate was 26 (95% confidence interval 23–29) per 10,000 person-years. Among women the incidence rate increased rapidly from the age of 50 and reached a peak of 119 per 10,000 person-years in women 80 years and older. The incidence rate among women 50 to 79 years old (56 per 10,000 person-years) was lower than that reported in previous studies of similar populations. Among men the incidence rate was low until the age of 80 years and older when it increased to 28 per 10,000 person-years. Fractures classified as AO type A comprised about 80% of the fractures in women and 64% in men. Almost two-thirds of all fractures were displaced and among men and women 80 years and older more than 80% of the fractures were displaced.

**Conclusion:**

The incidence rate of distal radius fracture in women 50 to 79 years old was lower than previously reported, which may indicate declining incidence in this group. In both sexes, the incidence was highest in the age group of 80 years and older. With a growing number of elderly in the general population, the impact of distal radius fracture in the future may be considerable.

## Background

The incidence of distal radius fracture has been studied frequently and shown to have increased over the years. In Sweden, the incidence rate in the city of Malmö had almost doubled between the time periods of 1953–1957 and 1980–1981 [1]. In the United States, a 17% increase in distal radius fractures was shown in Rochester, Minnesota, between 1945–1954 and 1985–1994 [2]. This change over time has been understood as a real increase in age-specific incidence rather than a result of an increase in diagnosed distal radius fractures [1]. Studies of fracture incidence in northern Europe have been carried out both on populations of larger cities such as Oslo and Bergen in Norway and Malmö in Sweden [1,3,4], and in whole counties such as Dorset in England, Fredriksborg in Denmark, and Uppsala in Sweden [5-7]. Studies have not shown statistically significant differences in the incidence rates of distal radius fracture in rural versus urban communities [8,9]. However, evidence of epidemiologic differences across Europe has been reported, with higher incidence rates of distal radius and other osteoporotic fractures in Scandinavia than in other European regions [10].

Since the epidemiology of distal radius fractures has changed during the last several decades, it is of interest to investigate whether the overall incidence is continuing to increase. Also, information about the incidence of different types of fracture is important because the type of fracture in terms of articular involvement and degree of displacement usually influences the choice of treatment and may impact the functional end-result.

Minimally displaced fractures of the distal radius are usually treated non-operatively while displaced fractures are treated either with closed reduction and immobilization with cast, percutaneous pinning or external fixation or, especially when intraarticular, with open reduction and internal fixation. Recently, the use of internal fixation for displaced fractures, which is probably the most costly and technically demanding treatment method, has been widely increasing. Thus, estimating the incidence of fractures classified according to articular involvement and fracture displacement would be of importance in determining costs and resource allocation for these injuries. Moreover, distal radius fractures may result in prolonged pain and functional impairment [11]. Complications such as persistent neuropathy of median, ulnar or radial nerve and fracture malunion have been reported in 1 out of 3 patients [12]. In this respect, fracture severity characteristics may be of importance. Previous Scandinavian studies of fracture incidence presented the proportions of fractures classified according to the methods of Older and Frykman [4,6,13,14]. The AO system (Arbeitsgemeinschaft fur Osteosyntesfrage) of fracture classification is being increasingly used in clinical studies of distal radius fracture as a measure of fracture severity [15]. To our knowledge there has been no published report regarding the incidence of acute distal radius fractures classified according to the AO system.

The aim of this investigation was to determinate the overall incidence of distal radius fractures and the age- and gender-specific incidence rates of the different types of distal radius fracture in the general population of Northeastern Scania in southern Sweden.

## Methods

The study was implemented in a representative population in Northeastern Scania, a region in the southern part of Sweden with an estimated total population of about 170,000 inhabitants. The region has 2 mid-size towns (Kristianstad and Hässleholm) and several smaller municipalities and their rural areas. The inclusion criteria for this study were acute fracture of the distal radius and age above 18 years. The exclusion criterion was person living outside the region according to the national population register at the time of fracture. In Northeastern Scania, persons with acute fractures seek medical attention mainly at the emergency department at Kristianstad Hospital. Besides, persons with minor fractures can be treated at the emergency department at Hässleholm Hospital. No other facility in the region manages distal radius fractures.

Persons with acute distal radius fracture were recorded prospectively at the two emergency departments during the period from January 1 through December 31, 2001. The fractures were registered by orthopedic surgeons or residents at the emergency department according to the International Classification of Diseases, Tenth Revision, Clinical Modification (ICD-10-CM) as S52.50, S52.51, S52.60 and S52.61. In addition to the persons identified through the emergency department register, inhabitants who had sustained fractures while traveling outside our defined study region were identified through review of a data register of our department's outpatient clinic where patients had scheduled visits after referral from other physicians or for follow-up. In order to identify persons with distal radius fracture who might have been given an incorrect diagnostic code at the emergency department we reviewed patient records for those who had received diagnostic codes for forearm fracture, wrist sprain or similar injuries. We also identified persons who had sustained a distal radius fracture at the hospital after being admitted for other reasons. All persons who undergo surgery are registered in another data register, and hereby we could double-check the data from the emergency department and also identify individuals who had been operated on but had not come through the emergency department. In this way all persons with diagnosed acute distal radius fracture in the population during the study period were probably identified.

All patient records were reviewed and date of injury, type of trauma and demographics were noted. Fall at the same level from an upright position was classified as moderate trauma and all other types of trauma (falling from heights, traffic accident or trauma during exercise) were classified as severe [4,5].

Standard posteroanterior and lateral radiographs were obtained to verify the diagnosis. At the conclusion of the study a single, experienced radiologist classified the fractures according to the AO-system and measured volar tilt and ulnar variance. Both interobserver reliability and intraobserver reproducibility for the AO classification have been shown to be fair when dividing the fractures into the different subgroups. When reducing the AO system to its three main types interobserver and intraobserver agreement were reported to be substantial [16]. Therefore we chose to classify the fractures into the 3 main AO types; type A is extraarticular, type B is partial articular and type C is complete intraarticular. Minimally displaced fractures were defined as volar tilt ranging from -5° to 20° and/or ulnar variance < 2 mm [17,18]. Fractures with greater displacement were defined as displaced.

The study was approved by the Regional Ethical Review Board.

### Statistical analysis

The incidence rates were calculated as the number of fractures divided by the mid-year population and expressed as incidence per 10,000 person-years. Mid-year population was calculated as the mean value of the population on December 31, 2000 and the population on December 31, 2001. Age- and gender- specific incidence rates were also calculated. The incidence rate was standardized to the Swedish general population using 10-year age groups (except for working-age population for which 5-year age groups were used).

Persons were divided into three age groups; 19 to 49 years, 50 to 79 years, and 80 years and older. Clinical factors were taken into consideration when the grouping was made; high-energy trauma is the common cause of distal radius fractures in young persons, whereas fractures after menopause are mostly related to osteoporosis. Among old age persons the level of function and comorbidity usually influence the choice of treatment [19]. In addition, it has been shown in previous studies that the incidence rate of distal radius fracture starts to markedly increase at the age of 50 years among women and 80 years among men [5,7,20].

Age- and gender-specific incidence rates were calculated for different types of fractures. The 95% confidence intervals (CI) for all incidence rates were calculated. The test of trends in Poisson rates was used to compare the overall incidence rates as well as the incidence of the different types of fractures among the three age groups; a p-value of less than 0.05 was considered to indicate statistical significance.

## Results

### Study population

During the 1-year period 427 persons were registered as having acute distal radius fracture. Sixty-five persons were excluded because they were living outside our defined study region. In addition, 27 persons were excluded because they were found not to have had an acute distal radius fracture. Hence, 335 persons with 340 fractures (5 simultaneous bilateral) were included (Table 1); 306 persons were treated at Kristianstad Hospital and 29 were treated at Hässleholm Hospital. Of the 335 persons, 311 were identified through the emergency department register, 15 were identified through the outpatient clinic register and 9 through the surgery register. Six persons were initially misclassified as having other diagnoses than distal radius fracture.

**Table 1:** Type of trauma and injured side among 335 persons older than 18 years with acute distal radius fracture in the region of Northeastern Scania, Sweden, during 2001

	Women	Men	Total
No. of persons with fractures (%)	261 (77.9)	74 (22.1)	335 (100)
Type of trauma, n (%)			
Moderate	191 (73.2)	35 (47.3)	226 (67.5)
Severe	67 (25.7)	38 (51.4)	105 (31.3)
Data missing	3 (1.1)	1 (1.4)	4 (1.2)
Side, n (%)			
Left	143 (54.8)	38 (51.3)	181 (54.0)
Right	116 (44.4)	31 (41.9)	147 (43.9)
Bilateral	1 (0.4)	4 (5.4)	5 (1.5)
Data missing	1 (0.4)	1 (1.4)	2 (0.6)

Percentages do not exactly sum to totals because of rounding

The mean age for women was 69 (range 19–101) years and the mean age for men was 55 (range 19–90) years. The trauma energy was moderate in nearly 70% of the fractures; trauma was severe in half of the men but in only one fourth of the women (Table 1). Among women 50 years and older the trauma energy was moderate in 177 (76%) and severe in 53 (23%). The trauma energy among men 50 years and older was moderate in 26 (57%) and severe in 19 (41%). Left-sided fractures were more common in both sexes.

### Incidence

**Figure 1:**
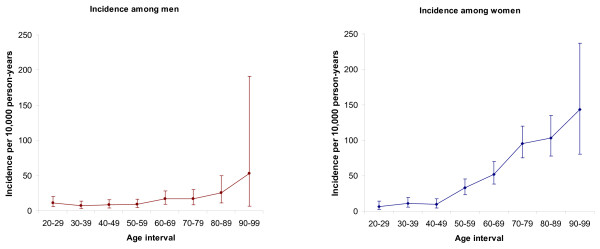
The age-specific incidence rates and 95% confidential intervals for distal radius fractures in the region of Northeastern Scania (Sweden) during 2001.

During 2001, the study region had a mid-year population of 129,094 inhabitants older than 18 years [21]. The overall incidence of distal radius fracture during 2001 was 26 (95% CI 23–29) per 10,000 person-years. Standardized to the Swedish general population the incidence rate was 24 (95% CI 22–27) per 10,000 person-years. Of the 335 persons with fracture, 261 were women, giving an incidence of 39 (95% CI 35–44) per 10,000 person-years. There were 74 fractures in men, giving an incidence of 12 (95% CI 9.2–14.7) per 10,000 person-years. The female:male ratio of the incidence rate of distal radius fracture was 3.3:1. The incidence increased with age in both men and women (Table 2). Below the age of 50 years the incidence was approximately 9 per 10,000 person-years irrespective of gender. Among women the incidence increased sharply from the age of 50 years and was almost doubling with each 10-year age interval to the age of 70 years and peaked after the age of 90 years to 144 per 10,000 person-years (Figure 1). The increase in incidence rate among women was highly significant when comparing the three age groups (p < 0.001). Among men the incidence remained low until the age of 80 years and older when it rose to 28 (95% CI 13.6–52) per 10,000 person-years (Table 2). Among men the difference in the incidence rate between any two of the three age groups was not statistically significant. However, when calculating the trend over all three age groups the increase was shown to be significant (p = 0.002).

**Table 2:** Number of persons with distal radius fractures, the population at risk, and the incidence per 10,000 persons in the region of Northeastern Scania, Sweden, during 2001

Sex	Age groups (year)	Population	No. of persons with fractures	Incidence	95% CI
Women	19–49	31547	28	8.9	5.9–12.8
	50–79	28132	158	56	48–66
	80-	6288	75	119	94–150
Men	19–49	32767	28	8.5	5.7–12.4
	50–79	26828	36	13.4	9.4–18.6
	80-	3532	10	28	13.6–52

CI = confidence interval

Among the population of working-age persons (19–65 years) the incidence rate was 13.4 (95% CI 11.2–15.9) per 10,000 person-years. Standardized to the Swedish general population aged 19–65 years, the incidence rate was 13.7 (95% CI 11.4–16.0) per 10,000 person-years. The incidence rate among working-age women was 17.2 (95% CI 13.7–21.2) per 10,000 person-years and among men was 9.7 (95% CI 7.2–12.9) per 10,000 person-years.

### Fracture AO classification

Radiographs of 8 persons were missing. In addition, classification according to the AO system was not possible in 5 fractures. Type-A fracture was the most common among all age groups, comprising 79% of the fractures in women and 64% in men (Table 3). The incidence of type C was low among women and men below the age of 50 years but increased with age and was highest in the age group of 80 years and older.

**Table 3:** Incidence of distal radius fractures per 10,000 persons in the region of Northeastern Scania, Sweden, during 2001, grouped according to AO type and age group

		19–49 years		50–79 years		80- years		
	AO type	n	Incidence (95% CI)	n	Incidence (95% CI)	n	Incidence (95% CI)	p-value*

Women	Type A	21	6.7 (4.1–10.2)	125	44 (37–53)	59	94 (71–121)	<0.001
	Type B	1	0.3 (0–1.8)	7	2.5 (1.0–5.1)	1	1.6 (0–8.9)	0.12
	Type C	4	1.3 (0.3–3.2)	21	7.5 (4.6–11)	13	21 (11–35)	<0.001
	Non-classifiable	2		1		0		
	Data missing	0		4		2		
Men	Type A	18	5.5 (3.3–8.7)	22	8.2 (5.1–12.4)	7	19.8 (8.0–41)	0.01
	Type B	5	1.5 (0.5–3.6)	3	1.1 (0.2–3.3)	0	0 (0–10.4)	0.56
	Type C	4	1.2 (0.3–3.1)	8	3.0 (1.3–5.9)	3	8.5 (1.8–25)	0.02
	Non-classifiable	1		1		0		
	Data missing	0		2		0		

* comparing incidence rates among the three age groups

### Fracture displacement

Almost two-thirds of all fractures were displaced (65% of the fractures in women and 61% in men). The incidence rates for minimally displaced and displaced fractures were similar below the age of 50 years (Table 4). The incidence of displaced fractures increased with age and, among women and men 80 years and older more than 80% of the fractures were displaced.

**Table 4:** Incidence of distal radius fractures per 10,000 persons in the region of Northeastern Scania, Sweden, during 2001, grouped according to fracture displacement and age group

		19–49 years		50–79 years		80- years		
	Fracture displacement	n	Incidence (95% CI)	n	Incidence (95% CI)	n	Incidence (95% CI)	p-value*

Women	Minimally displaced	16	5.1 (2.9–8.2)	55	19.6 (14.7–25)	13	21 (11–35)	p < 0.001
	Displaced	11	3.5 (1.7–6.2)	99	35 (29–43)	60	95 (73–123)	p < 0.001
	Data missing	1		4		2		
Men	Minimally displaced	15	4.6 (2.6–7.6)	12	4.5 (2.3–7.8)	0	0 (0–10.4)	P = 0.52
	Displaced	13	4.0 (2.1–6.8)	22	8.2 (5.1–12.4)	10	28 (13.6–52)	p < 0.001
	Data missing	0		2		0		

* comparing incidence rates among the three age group

## Discussion

This study showed that the incidence of distal radius fracture among women was increasing with age while among men it remained low until old age. Our observations differ from previous studies from Norway [3,4] and the United Kingdom [22] in which the incidence rate among women increased after menopause and then tended to level off or plateau from the age of 60 years. However, our findings are in agreement with other Scandinavian and British studies [1,2,7,20] that also reported that the incidence among women was increasing with age. There is no convincing explanation to the described plateau among postmenopausal women. Some authors have suggested that the plateau or decrease could be due to age-related decreases in speed and strength of extending the arm to protect other parts of the body during falls [23]. It is not clearly understood how this explanation relates to the incidence pattern seen in this and other studies. A steady rise in fracture incidence after menopause may reflect osteoporosis and increased risk of falling as the main predictors of fracture in the older female population. The findings in our study that the trauma energy was moderate in 3 of 4 women 50 years and older and that the incidence of displaced fractures was highest among the oldest women may reflect that low bone density increases the risk of fracture. In addition, Davies et al. reported that the risk of falling increased after menopause among women, possibly due to poor reaction time and reduced muscle strength [24].

We showed an overall incidence rate of 26 per 10,000 person-years, which did not change substantially when standardized to the Swedish general population. Our data suggest that, each year in Sweden, up to 18,000 persons above 18 years of age will sustain a distal radius fracture. According to our findings, the standardized incidence rate among the population of working-age persons (19 to 65 years) was 14 per 10,000 person-years. The incidence rate was almost twice as high among women as among men in this age group. With an increasingly cost conscious management of health care, the economic and social burden of distal radius fracture should be of relevance and needs further study.

**Figure 2:**
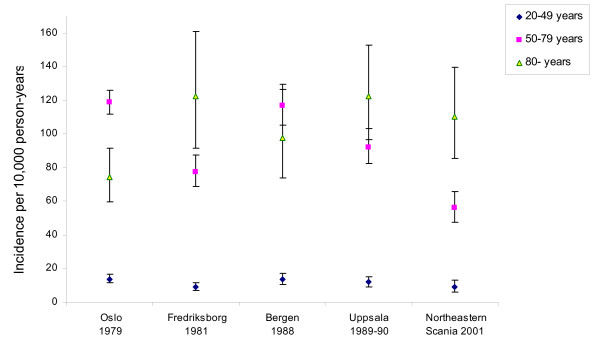
The age-specific incidence rates and 95% confidence intervals for distal radius fractures among women in Oslo (Norway), Bergen (Norway), Fredriksborg (Denmark), Uppsala (Sweden) and Northeastern Scania (Sweden) [3–6].

Our study showed a lower incidence rate among women 50–79 years than previously reported in studies from Bergen and Oslo in Norway, Fredriksborg in Denmark, and Uppsala in Sweden [3-6] (Figure 2). This might indicate a real decline in the incidence of distal radius fracture, but other factors could have influenced our results. The overall incidence rate in Northeastern Scania was considerably lower than the incidence rate of 38 per 10,000 person-years in the Norwegian city of Bergen, reported by Hove et al. [4]. This, however, is to our knowledge the highest reported incidence of distal radius fracture. Possible causes to this difference in reported incidence pattern include factors affecting tendency to fall and prevalence of osteoporosis. Weather conditions during the study periods also may have influenced the results of incidence rates [3-5]. In our study region there has been an ongoing project for osteoporosis since 1994 with information to primary care physicians about the importance of identifying patients with low bone mineral density and offering them medical treatment. When comparing the incidence rates reported by different studies it is necessary to be cautious because of the variability in data collection methods and study design. In our study as well as in others [4,5,5,14,20] the persons were recorded prospectively, but several studies were retrospective [1-3,22]. Further, the inclusion criteria were based on radiographs in some reports [4-6] and on patient records in others [3,22]. The incidence rate was reported for 5-year age groups in some studies [5,7,20,22] and 10-year age groups in others [1,3,4,6]. We did not attempt to compare reported incidence rates for men because the small number of fractures would give insufficient precision.

Two previous Scandinavian epidemiologic studies have reported results of incidence of different types of fractures classified according to the method of Older [4,6]. Non-displaced fractures (Older type I) were most common among postmenopausal women according to both Hove et al. and Solgaard et al. The number of displaced fractures was highest among women in the age groups of 60 to 69 years in Bergen [4] and 70 to 79 years in Fredriksborg [6]. Hove et al. showed that, among men, the degree of displacement was similar in all age groups. Solgaard et al. found that the proportion of non-displaced fractures was high in young men, whereas among the older men the four Older types of fracture were equally distributed. Classification according to Frykman was used in population studies in Stockholm [14] and on Iceland [13]; intraarticular fractures (Frykman types 3 to 8) comprised 33 percent of the fractures in Stockholm and 52 percent on Iceland. Bengnér et al. defined fractures that were reduced as displaced fractures and showed that the number of reduced fractures increased with age [1].

In the present study we found that extraarticular (type A) fracture was the most common type among both men and women and that the incidence increased with age. The incidence rate of minimally displaced fractures was slightly higher than that of displaced fractures among the youngest age groups of men and women but displaced fractures were twice as common as minimally displaced fractures among the 50 to 79 years age group. The incidence of displaced fractures was much higher than that of minimally displaced fractures among women and men 80 years and older. All together, almost two-thirds of the fractures in our study were displaced.

This study may have some limitations. The data were drawn from a relatively small community and hereby a limited number of persons with acute distal radius fracture. This could have affected the precision of our estimates. However, the population is well defined and considering the multiple steps in our case identification process the possibility that we failed to include a substantial number of persons with acute distal radius fracture in our study region would be very small. Of all persons who had received a diagnostic code for distal radius fracture at the emergency department, 27 persons were excluded because, on reviewing all patient records, they were found not to have had an acute distal radius fracture. These persons had accordingly been misclassified at the emergency department and can be considered as "false positives". Theoretically, some persons who had a distal radius fracture could in the same way have been misclassified as having had other diagnoses, i.e. "false negatives". However, all patients who were treated with surgery or with closed reduction and cast were followed-up at our outpatient clinic. Because we reviewed the outpatient clinic and the surgery data registers, we could hereby double-check and include eligible persons who had initially been missed or misclassified at the emergency department. We also reviewed patient records for those who had received other traumatic wrist and forearm diagnoses at the emergency department to detect any incorrectly coded distal radius fractures. Some persons with minimally displaced fractures who were treated with cast only may have been followed-up outside our clinic. It is possible that this group of persons may hide a number of "false negatives" that were misclassified and that were not detected in our patient record review. However, this number ought to be very small and should not have substantially influenced our incidence results.

The radiographic assessment was done by a single radiologist, which may have influenced the reliability of the classification regarding AO type and displacement. However, the AO classification has been shown to have good intraobserver reliability when restricted to the 3 main AO types and a possible minor degree of misclassification of displacement should not have a substantial impact on the results.

## Conclusion

In our study the incidence rate of distal radius fracture increased with age in both women and men. Extraarticular (AO type A) fracture was the most common type of fracture in all age groups. Almost two-thirds of the fractures were displaced. Our finding of a lower incidence rate among women aged 50 to 79 years than previously reported in similar populations might indicate a real decrease in incidence of distal radius fracture in this group. The incidence rate was highest among persons 80 years and older. Thus, even if the age- and gender-specific incidence does not continue to rise, the impact of distal radius fractures in the future will be important because of the increasing number of elderly in the general population.

## Competing interests

The author(s) declare that they have no competing interests.

## Authors' contributions

EB contributed to study conception, analysis and interpretation of data and drafting of the manuscript; IA participated in study conception and design and critical revision of the manuscript; MP participated in acquisition of data. All authors read and approved the final manuscript.

## Pre-publication history

The pre-publication history for this paper can be accessed here:


